# Infection and injury cooperatively induce a tissue resident adaptive NK cell program that propagates lung damage and mortality

**DOI:** 10.21203/rs.3.rs-10686332/v1

**Published:** 2026-10-26

**Authors:** Daniel Calabrese, Reena Bharti, Natalie Mosqueda, Dongliang Zhang, Longhui Qiu, Francesca Lunardi, Annika Schmidt, Avishai Shemesh, Jonathan Singer, Steven Hays, Oscar Aguilar, Mark Looney, John Greenland

## Abstract

Cytomegalovirus (CMV) is a herpesvirus that persists latently after acute infection and reactivates during periods of stress. CMV infection remains a major driver of morbidity in solid organ transplant recipients, and how it worsens transplant outcomes is unknown. In 669 lung transplant recipients, latent donor CMV and primary graft dysfunction (PGD), a severe form of acute lung injury, synergistically promoted CMV reactivation, worse gas exchange, and mortality. In mice, ischemia-reperfusion injury reactivated latent CMV and drove durable expansion of tissue-resident, adaptive-like natural killer (NK) cells. Adoptive transfer of CMV-educated Ly49H⁺ NK cells was sufficient to induce acute lung injury. Mortality tracked this NK cell program rather than viral load. Depleting NK cells prevented injury and rescued survival, whereas suppressing viral replication with ganciclovir did not. While CMV clinical trials to date have targeted suppression of viral replication, these findings reframe CMV-associated injury as an NK cell-driven process and identify the local adaptive NK compartment as a tractable therapeutic target.

